# Evolution of the *Staphylococcus argenteus* ST2250 Clone in Northeastern Thailand Is Linked with the Acquisition of Livestock-Associated Staphylococcal Genes

**DOI:** 10.1128/mBio.00802-17

**Published:** 2017-07-05

**Authors:** Danesh Moradigaravand, Dorota Jamrozy, Rafal Mostowy, Annaliesa Anderson, Emma K. Nickerson, Janjira Thaipadungpanit, Vanaporn Wuthiekanun, Direk Limmathurotsakul, Sarunporn Tandhavanant, Chanthiwa Wikraiphat, Gumphol Wongsuvan, Nittaya Teerawattanasook, Yaowaruk Jutrakul, Nuttiya Srisurat, Prajuab Chaimanee, T. Eoin West, Beth Blane, Julian Parkhill, Narisara Chantratita, Sharon J. Peacock

**Affiliations:** a Wellcome Trust Sanger Institute, Wellcome Genome Campus, Hinxton, Cambridgeshire, United Kingdom; b Department of Infectious Disease Epidemiology, School of Public Health, Imperial College London, London, United Kingdom; c Pfizer Vaccine Research and Development, Pearl River, New York, USA; d Cambridge University Hospitals NHS Foundation Trust, Cambridge, United Kingdom; e Mahidol-Oxford Tropical Medicine Research Unit, Faculty of Tropical Medicine, Mahidol University, Bangkok, Thailand; f Department of Microbiology and Immunology, Faculty of Tropical Medicine, Mahidol University, Bangkok, Thailand; g Department of Clinical Pathology, Sunpasitthiprasong Hospital, Ubon Ratchathani, Thailand; h Department of Clinical Pathology, Udon Thani Regional Hospital, Udon Thani, Thailand; i Department of Clinical Pathology, Khon Kaen Regional Hospital, Khon Kaen, Thailand; j Srinagarind Hospital, Faculty of Medicine, Khon Kaen University, Khon Kaen, Thailand; k Division of Pulmonary and Critical Care Medicine, Department of Medicine, University of Washington, Seattle, Washington, USA; l International Respiratory and Severe Illness Center, University of Washington, Seattle, Washington, USA; m Department of Medicine, University of Cambridge, Cambridge, United Kingdom; n London School of Hygiene and Tropical Medicine, London, United Kingdom; Emory University School of Medicine; Emory University

**Keywords:** genomic epidemiology, *Staphylococcus argenteus*, *Staphylococcus aureus*, antibiotic resistance

## Abstract

*Staphylococcus argenteus* is a newly named species previously described as a divergent lineage of *Staphylococcus aureus* that has recently been shown to have a global distribution. Despite growing evidence of the clinical importance of this species, knowledge about its population epidemiology and genomic architecture is limited. We used whole-genome sequencing to evaluate and compare *S. aureus* (*n* = 251) and *S. argenteus* (*n* = 68) isolates from adults with staphylococcal sepsis at several hospitals in northeastern Thailand between 2006 and 2013. The majority (82%) of the *S. argenteus* isolates were of multilocus sequence type 2250 (ST2250). *S. aureus* was more diverse, although 43% of the isolates belonged to ST121. Bayesian analysis suggested an *S. argenteus* ST2250 substitution rate of 4.66 (95% confidence interval [CI], 3.12 to 6.38) mutations per genome per year, which was comparable to the *S. aureus* ST121 substitution rate of 4.07 (95% CI, 2.61 to 5.55). *S. argenteus* ST2250 emerged in Thailand an estimated 15 years ago, which contrasts with the *S. aureus* ST1, ST88, and ST121 clades that emerged around 100 to 150 years ago. Comparison of *S. argenteus* ST2250 genomes from Thailand and a global collection indicated a single introduction into Thailand, followed by transmission to local and more distant countries in Southeast Asia and further afield. *S. argenteus* and *S. aureus* shared around half of their core gene repertoire, indicating a high level of divergence and providing strong support for their classification as separate species. Several gene clusters were present in ST2250 isolates but absent from the other *S. argenteus* and *S. aureus* study isolates. These included multiple exotoxins and antibiotic resistance genes that have been linked previously with livestock-associated *S. aureus*, consistent with a livestock reservoir for *S. argenteus*. These genes appeared to be associated with plasmids and mobile genetic elements and may have contributed to the biological success of ST2250.

## IMPORTANCE

In this study, we used whole-genome sequencing to understand the genome evolution and population structure of a systematic collection of ST2250 *S. argenteus* isolates. A newly identified ancestral species of *S. aureus*, *S. argenteus* has become increasingly known as a clinically important species that has been reported recently across various countries. Our results indicate that *S. argenteus* has spread at a relatively rapid pace over the past 2 decades across northeastern Thailand and acquired multiple exotoxin and antibiotic resistance genes that have been linked previously with livestock-associated *S. aureus*. Our findings highlight the clinical importance and potential pathogenicity of *S. argenteus* as a recently emerging pathogen.

## INTRODUCTION

Until recently, *Staphylococcus argenteus* was considered a community-associated lineage of *Staphylococcus aureus* and was classified by multilocus sequencing typing (MLST) as clonal complex 75 (CC75) (1, 2). However, multiple lines of evidence, including genetic distance from *S. aureus*, supported its reclassification as a distinct species (2–4). *S. argenteus* was first reported in northern Australia (5), and early descriptions were connected with remote communities, but an increasing number of reports have confirmed that this species is globally distributed, with most reports originating in tropical areas (2, 6, 7). CC75 is composed of four sequence types (STs) that have been isolated in various European and Far Eastern countries (2, 4, 6). One of the most frequent STs is ST2250, which has been isolated in the United Kingdom and from patients with community onset invasive infections in several provinces in Thailand (7, 8).

*S. argenteus* has been proposed to be a less pathogenic ancestral lineage of *S. aureus* (9). *S. argenteus* is less resistant to a range of antibiotics than *S. aureus* is and lacks some well-characterized *S. aureus* virulence factor genes. This includes an apparently universal absence of staphyloxanthin (a carotenoid pigment that protects against oxidative stress [9]) and an absence of the gene encoding Panton-Valentine leukocidin in the majority of isolates (5, 6, 9, 10). Isolation in Australia has predominantly been in the context of skin and soft tissue infections (5), but *S. argenteus* has been proposed to be an important cause of community-acquired invasive infections in Thailand, where a sharp rise in its prevalence has been reported since 2006 and 2007 (8). A large multicenter study in Thailand in which the clinical features of patients with invasive infections caused by *S. argenteus* and *S. aureus* were compared found that rates of bacteremia and drainage procedures were similar in the two groups (8). *S. argenteus* precipitated significantly less respiratory failure than *S. aureus*, with a similar but nonsignificant trend for shock, but this did not translate into a difference in death at 28 days (8). This suggests that *S. argenteus* is equipped with genes that facilitate invasion of and virulence in humans.

*S. argenteus* harbors a smaller accessory genome than *S. aureus* but has a genome with several distinctive features (9). The genome of *S. argenteus* MSHR1132 includes a set of clustered regularly interspaced short palindromic repeat (CRISPR) elements, which are rare in *S. aureus*. This suggests that changes in the genetic repertoire of *S. argenteus*, including the acquisition of accessory genes, may be affected by mechanisms different from those seen in *S. aureus* (9). This, together with the reported lack of recombination between *S. argenteus* and *S. aureus*, is consistent with the genetic separation of *S. argenteus* (9). However, since that study was limited to one *S. argenteus* genome, the conclusions are not generalizable.

To provide detailed population and epidemiological genomic insights into *S. argenteus*, we performed whole-genome sequencing of a systematic collection of 68 *S. argenteus* (predominately ST2250) and 251 *S. aureus* isolates from two previous studies in which isolates were recovered from patients with community-associated invasive infections at multiple hospitals across northeastern Thailand (8). We demonstrate that the ST2250 clone has spread across northeastern Thailand over the last few decades. Our results confirm a distinctive profile of antibiotic resistance genes in *S. argenteus* and identify genes that are exclusive to ST2250, including multiple exotoxin and tetracycline resistance genes, some of which have been associated previously with livestock-associated *S. aureus*.

## RESULTS

**FIG 1:**
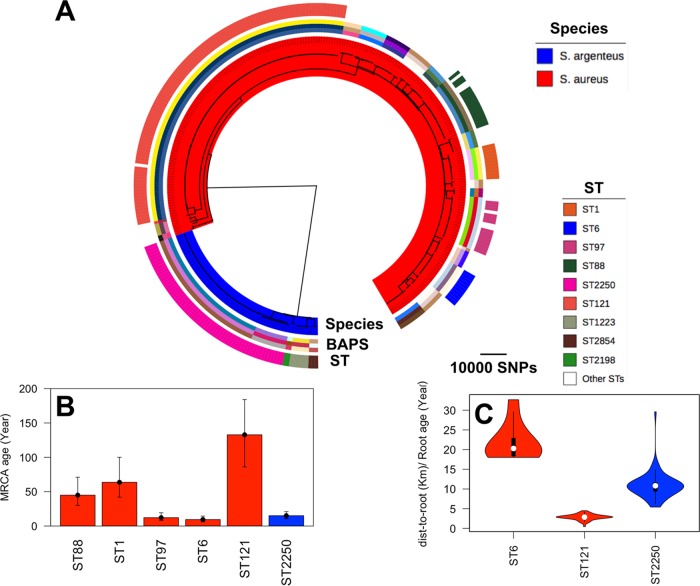
(A) Phylogenetic tree constructed from SNPs in the *S. aureus* (red) and *S. argenteus* (blue) core genome alignment. Outer ring: STs of clusters containing 10 or more isolates. Inner ring: BAPS clustering represented by three bands to depict three parameter values, i.e., 20, 40, and 60, of the estimated number of clusters. (B) Bayesian analysis to define the age of the MRCA of *S. argenteus* ST2250 and the five predominant *S. aureus* STs (ST88, ST1, ST97, ST6, and ST121). Error bars denote 95% CIs (see Materials and Methods for details). (C) Distribution of rates of spread of ST2250, ST121, and ST6, which had a temporal signal. Each box shows the interquartile range, and the whiskers indicate the boundary of 1.5 times the interquartile range. The white marker denotes the median value. The colored area is the probability density of the data at different values.

The 251 *S. aureus* and 68 *S. argenteus* isolates used in this study originated from patients with invasive staphylococcal diseases who resided across northeastern Thailand. The *S. argenteus* population was composed mainly of ST2250 (57/68, 83%), and the rest were assigned to ST1223, ST2854, and ST2198 (Fig. 1A). All four *S. argenteus* STs have been isolated in other countries, indicating that the *S. argenteus* isolates obtained in Thailand belong to globally circulating lineages. The *S. aureus* collection was more diverse, but five STs (ST121, ST88, ST1, ST97, and ST6) each contained at least 10 isolates and together constituted 63% of the *S. aureus* collection. The predominant *S. aureus* ST (ST121, 108 isolates) has been identified previously as common in Thailand and other Far Eastern and European countries (7, 28–30). Isolates within the major *S. aureus* and *S. argenteus* ST clades originated in hospitals across the region. ST2250, ST121. and ST88 contained isolates from all four hospitals, whereas ST1, ST6, and ST97 were from three hospitals (see Fig. S1A in the supplemental material).

10.1128/mBio.00802-17.1 FIG S1  (A) Distribution of major *S. argenteus* and *S. aureus* STs across hospitals. (B) Pairwise geographical distances versus pairwise SNP distances for isolates in major STs. Download FIG S1, PDF file, 0.4 MB. Copyright © 2017 Moradigaravand et al. 2017 Moradigaravand et al. This content is distributed under the terms of the Creative Commons Attribution 4.0 International license.

The phylogenetic tree reconstructed from the single nucleotide polymorphisms (SNPs) in the core genome shared by *S. argenteus* and *S. aureus* revealed a clear distinction between the two species, together with a clonal structure within each species (Fig. 1A). The population structure inferred by Bayesian analysis was highly concordant with STs for *S. aureus*, while *S. argenteus* ST2250 consisted of two closely related Bayesian analysis of population structure (BAPS) clusters (Fig. 1A). Mapping of patient residences has been reported previously for all but 10 cases and showed that patients infected with *S. argenteus* and *S. aureus* were drawn from a comparable geographic area across northeastern Thailand (7, 8). To determine whether patients with genetically related isolates were spatially clustered, the geographic distance was plotted against the pairwise SNP distance of each of the major *S. argenteus* and *S. aureus* clades. This demonstrated low geographical clustering, with isolates with the same SNP distances, including closely related isolates, being uniformly distributed across the region (Fig. S1B).

The genomic characteristics of *S. argenteus* ST2250 (based on 2,107 core genome SNPs) and the five major STs in the *S. aureus* population (ST121, ST88, ST1, ST6, and ST97, containing 7,424, 1,852, 1,809, 931, and 1,201 SNPs, respectively) were compared (Fig. S2). The pairwise SNP distance of isolates within the *S. argenteus* ST2250 clade indicated high diversity, with few instances of closely related isolates in the major *S. argenteus* and *S. aureus* clades (Fig. S2A to C). For instance, only 11 isolate pairs (average difference of 11 SNPs) of 2,211 isolate pairs were <20 SNPs apart (Fig. S2A), suggesting that the collection does not contain isolates associated with one or more outbreaks.

10.1128/mBio.00802-17.2 FIG S2  (A) Pairwise SNP distance distribution, root-to-tip distance versus year of isolation, distribution of R-squared values from the resampling test (dashed lines show real R-squared values), and Bayesian trees for isolates in ST2250 and ST6 in the Thai population. Values on the nodes are ages, and bars denote 95% CIs. (B) Pairwise SNP distance distribution, root-to-tip distance versus year of isolation, distribution of R-squared values from the bootstrapping test (dashed lines show real R-squared values), and Bayesian trees for the ST121 clade within Thailand with and without global isolates, as detailed in the text. Values on the nodes are node ages, and bars denote 95% CIs. (C) Pairwise SNP distance distribution and root-to-tip distance versus year of isolation for isolates in clades without a temporal signal. Download FIG S2, PDF file, 0.5 MB. Copyright © 2017 Moradigaravand et al. 2017 Moradigaravand et al. This content is distributed under the terms of the Creative Commons Attribution 4.0 International license.

Bayesian analysis indicated a substitution rate of 4.66 (95% confidence interval [CI], 3.12 to 6.38) mutations per genome per year (or 1.76 × 10^−6^ per site per year) for *S. argenteus* ST2250. This was comparable to the substitution rate of 3.53 (95% CI, 2.87 to 4.18) for *S. aureus* ST121 in our collection, which is slightly lower than a previous estimate of 5.6 (95% CI, 3.36 to 7.84) for a global collection of CC121, including ST121 (13). The age of the most recent common ancestor (MRCA) of ST121 in our collection was 132 (95% CI, 86 to 184) years, which is comparable to the previously estimated MRCA of 129 (95% CI, 88 to 186) years in reference 13. Our results indicate that *S. argenteus* ST2250 emerged in Thailand an estimated 15 years ago, as did *S. aureus* ST97. However, the *S. aureus* ST1, ST88, and ST121 clades emerged around 100 to 150 years ago (Fig. 1B). Moreover, *S. aureus* ST121 from Thailand was only distantly related to ST121 isolates from Europe and Africa, with a >150-year divergence between Thai and non-Thai isolates (Fig. S2B). The ancestral clade appeared to have undergone a subsequent expansion over a period of 40 to 60 years (Fig. S2B).

*S. argenteus* ST2250 appeared to have been geographically disseminated across northeastern Thailand at a higher rate than *S. aureus* ST121 (Fig. 1C and S2A). To take into account the fact that ST121 is an old clade, we considered the rate of geographical expansion of the subclade that formed 58 years ago as shown in Fig. S2B. The ST6 clade appeared to be more recent than the ST2250 clade and has spread at a higher rate than ST2250 (Fig. 1C). These findings indicate that, compared to most *S. aureus* clones, *S. argenteus* ST2250 is a relatively recent clone that is spreading rapidly in northeastern Thailand.

**FIG 2:**
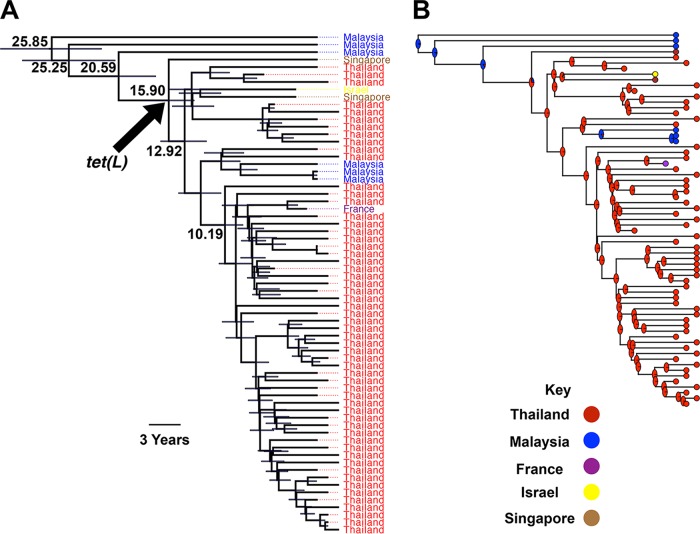
(A) Bayesian phylogenetic tree of *S. argenteus* ST2250 including isolates from Thailand and elsewhere. Node values are node ages in years. Bars on the nodes show 95% CIs. The arrow shows the node in which the *tet*(L) gene was acquired. (B) Phylogenetic tree of the data presented in panel A with ancestral states of nodes inferred from maximum-likelihood analysis. Pie charts show the marginal probability of each status (country of origin) for each node.

The dissemination of ST2250 in Thailand may be associated with a broader global circulation of ST2250. To explore this, we compared the Thai genomes with those of *S. argenteus* isolates recovered in Malaysia, Singapore, Israel, and France. The resulting tree indicated that the four Malaysian and Singaporean isolates were ancestral to the expansion of ST2250 in Thailand. The introduction of this lineage into Thailand appeared to have occurred within the past 20 to 30 years (Fig. 2A). After this putative introduction, the Thai lineage served as the source of reintroductions into Malaysia and Singapore, as well as France and Israel on at least three occasions, indicating regional and more distant transmissions (Fig. 2A and B). The inferred Malaysian status of the root of the ST2250 clade was observed when the analysis was repeated for different subsamples of Thai isolates, and therefore, the findings do not appear to be influenced by the different sizes of the Thai and non-Thai collections (Fig. S3A). We also observed mixing between Thai ST2250 and non-Thai isolates for randomly generated collections of Thai and non-Thai isolates. Furthermore, to account for the temporal bias in sample collection, we repeated the analysis for the collection composed of non-Thai and Thai isolates that were collected in the same period as non-Thai isolates, i.e., between 2009 and 2011 (Fig. S3B). The resulting tree indicated that the findings for the status of the root and mixing of isolates remained robust (Fig. S3B).

10.1128/mBio.00802-17.3 FIG S3  (A) Mean marginal likelihood values of the inferred Malaysian status of the ancestor of the ST2250 clade for 50 samples set composed of non-Thai isolates and 5, 7, 10, 15, 20, and 25 randomly selected Thai isolates. The error bars show the standard deviation for each sample set. (B) Phylogenetic tree with the ancestral states of the sample set composed of ST2250 non-Thai and Thai isolates that were isolated in the same period as the non-Thai isolates, i.e., between 2009 and 2011. Download FIG S3, PDF file, 0.1 MB. Copyright © 2017 Moradigaravand et al. 2017 Moradigaravand et al. This content is distributed under the terms of the Creative Commons Attribution 4.0 International license.

**FIG 3:**
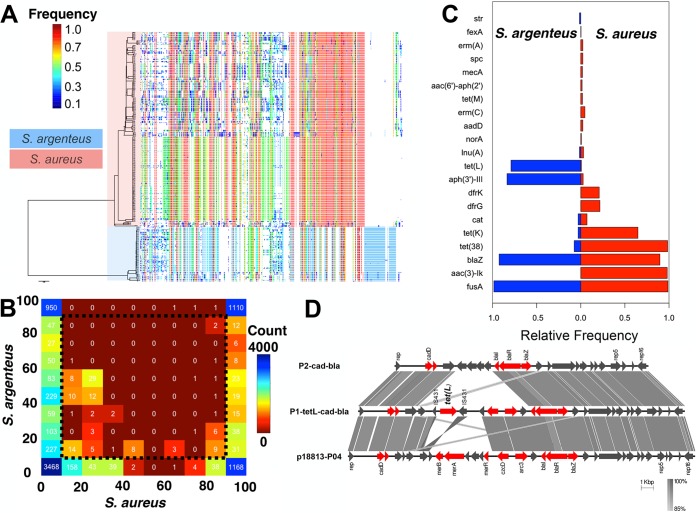
(A) Phylogenetic tree based on SNPs in the core genome of *S. aureus* and *S. argenteus* and the presence or absence of accessory genes across the genomes. Heatmap colors denote the frequency of each accessory gene across the collection. (B) Comparative pangenome analysis of *S. aureus* and *S. argenteus*. The values on each side of the table show the percentages of isolates of that species harboring the gene, and the values within the table are the numbers of genes shared by those isolates. (C) The relative frequency of known antibiotic resistance genes in the *S. argenteus* and *S. aureus* populations. (D) Alignment of genomic components of plasmids P1-tetL-cad-bla and P2-cad-bla, and known plasmid p18813-P04.

We then compared the genomes of *S. argenteus* and *S. aureus* to determine the extent to which the two collections share core and accessory genes (Fig. 3A). A total of 1,015 genes were present in every *S. aureus* and *S. argenteus* isolate. Using a lower identity cutoff of 70% to define a locus match, Méric et al. found 1,478 genes present in every isolate of *S. aureus* and *S. epidermidis* (14), which is more distantly related to *S. aureus* than *S. argenteus* is. Although we used a different pangenome construction method, repetition of our analysis with a 70% identity cutoff for a locus match identified 1,813 genes. This reveals that the closer genetic distance is also reflected in a greater proportion of shared genes.

Defining the core genome as the genes present in >90% of the isolates examined, we found a total of 1,110 genes in the shared core genome of *S. argenteus* and *S. aureus*. The numbers of core genes identified in *S. argenteus* and *S. aureus* were 2,063 and 2,412, respectively. This means that around half of the genes of each species are exclusive, indicating a high level of divergence and strong support for their classification as separate species (15) (Fig. 3A and B). The numbers of accessory genes (present in <90% of the isolates) of *S. argenteus* and *S. aureus* were comparable (5,977 and 5,651, respectively), indicating that the accessory genome of *S. argenteus* is equivalent in size to that of *S. aureus*. This contrasts with the previous report on *S. argenteus* MSHR1132, where a single genome was analyzed (9). As shown in Fig. 3B, the two species shared some genes that were otherwise variably present in the two populations. Genes present in <90% and >10% of the isolates of both species might be involved in ongoing adaptation and so were further explored. The majority of the genes in this category were associated with phages. Excluding phage-related proteins, several putative virulence genes were detected that were associated with known phages. These included the staphylokinase encoded by *sak*, which increases bacterial resistance to the host immune response (16). This gene had been independently acquired by several lineages of both species (Fig. S4 and S5) and appeared to be carried by the temperate Sa3int phage (17) present in the majority of ST2250 and other *S. argenteus* isolates (Fig. S4). Several putative pathogenicity island genes, e.g., group_448, group_1635, and group_1289 genes, were detected in ST2250 isolates and in some *S. aureus* STs (particularly ST121) (Fig. S4), and the linked bacteriophage encoding Panton-Valentine leukocidin (*pvl*) was sporadically distributed in *S. argenteus* ST2250 but was predominant in *S. aureus* ST121 (Fig. S4 and S5). Recombinant regions in ST2250 were limited to a small phage region in the genome, although the inserted elements appeared to be similar to *S. aureus* genomic regions in the NCBI nonredundant nucleotide database. These results reveal similarities between putative pathogenicity mechanisms and bacteriophages in both species.

10.1128/mBio.00802-17.4 FIG S4  Distribution of selected accessory genes present in >10 and <90% of the isolates in the *S. argenteus* and *S. aureus* collection across the phylogeny. The red and light blue backgrounds of clades denote *S. aureus* and *S. argenteus*, respectively. Horizontal bars show gene presence (red) or absence (blue). Phage-related genes and genes with no annotated function were excluded. Download FIG S4, PDF file, 0.1 MB. Copyright © 2017 Moradigaravand et al. 2017 Moradigaravand et al. This content is distributed under the terms of the Creative Commons Attribution 4.0 International license.

10.1128/mBio.00802-17.5 FIG S5  Distribution of mobile genetic elements and plasmids across the *S. argenteus* phylogeny. Yellow and gray backgrounds of clades represent ST2250 isolates containing plasmids P1-tet(L)-cad-bla and P2-cad-bla, respectively. Download FIG S5, PDF file, 0.1 MB. Copyright © 2017 Moradigaravand et al. 2017 Moradigaravand et al. This content is distributed under the terms of the Creative Commons Attribution 4.0 International license.

The observation that ST2250 is a recently expanded lineage led us to investigate accessory genes that were specific to this lineage and might account for its biological success. Several gene clusters were present in ST2250 isolates but absent from the other *S. argenteus* and *S. aureus* isolates (Fig. S6A and B). These included an enterotoxin gene cluster composed of *seC-bov* (enterotoxin C-bovine) and *entQ* (staphylococcal enterotoxin Q). These were inserted into the chromosome upstream of a region that was identified as a putative vSaβ genomic island, which may indicate that these are accessory components of the vSaβ island. vSaβ and vSaα are genetic elements identified in various *S. aureus* genomes that contain a number of variably distributed putative virulence genes (18). Both genetic elements were identified in *S. argenteus* MSHR1132, suggesting that the islands were likely acquired by a common ancestor of *S. aureus* and *S. argenteus* (9). Sequences flanking the *seC-bov/entQ* gene cluster were homologous with the *S. argenteus* MSHR1132 genome, suggesting that the cluster was inserted into a conserved genomic region. The coding sequence identified immediately downstream of *sec-bov* was highly similar (94% nucleotide sequence identity) to MSHR1132 locus SAMSHR1132_16480, located within vSaβ. Furthermore, a sequence corresponding to *entQ* and *sec-bov* together with flanking genes was identified in the genomes of various human clinical *S. aureus* isolates, e.g., ST772-MRSA-V (GenBank accession number CP010526), which means that gene sharing by the two species might have occurred via homologous recombination. The presence of pathogenicity genes associated with lineages of bovine origin implies links between *S. argenteus* and livestock-associated *S. aureus*. Furthermore, we also found several linked exotoxin genes (i.e., group_5536 to group_5540, group_5545, and group_5546 in Fig. S6) seemingly located in an operon and lipoprotein genes, all of which were found on the vSaα genomic island, which shares high sequence identity with the corresponding chromosome region of MSHR1132 (96% sequence identity over 92% of the sequence length) (Fig. S6A and B). We also identified a CRISPR element exclusively in the ST2250 clade that is known to be involved in defense against mobile genetic elements (19, 20) that was linked to *hsd* and *cas* genes and inserted into *orfX*, as described in reference 9 (Fig. S6A and B). However, in contrast to the MSHR1132 reference genome, no staphylococcal cassette chromosome *mec* (SCC*mec*) element was associated with these genes. This supports the hypothesis that these CRISPR elements are highly mobile and capable of horizontal transfer, even independently of SCC*mec* (9). These elements are rare in human-associated *S. aureus*, and only livestock-associated *S. aureus* ST398 has been previously reported to harbor a CRISPR-Cas locus (21). Taken together, our results indicate that genes specific to *S. argenteus* ST2250 and absent from the *S. aureus* isolates may have originated from other ancestral isolates and are linked to livestock-associated lineages.

10.1128/mBio.00802-17.6 FIG S6  (A) Genes exclusive to ST2250 (absent from the other *S. argenteus* and *S. aureus* clades in the study collection). Groups of genes referred to in the text are red, purple, and green. (B) Alignment of vSaα in one study isolate (green genes in panel A) and the published pathogenicity island of *S. argenteus* MSHR1132. Download FIG S6, PDF file, 0.2 MB. Copyright © 2017 Moradigaravand et al. 2017 Moradigaravand et al. This content is distributed under the terms of the Creative Commons Attribution 4.0 International license.

*S. argenteus* is known to have lower phenotypic resistance to antimicrobial drugs than *S. aureus* (8). We extended this previous analysis by defining the relative frequency of genes encoding resistance to commonly used antimicrobial drugs (Fig. 3C) and defining the relationship between phylogeny and phenotypic (detailed in reference 8) or genetic resistance (Fig. S7). *blaZ* accounted for penicillin resistance in the two collections (Fig. S7A), but the *blaZ* variant in *S. argenteus* ST2250 differed from that in ST121 and was more similar to those in uncommon STs in the *S. aureus* collection (Fig. S7B). Tetracycline resistance genes varied in the two species (Fig. 3C, S5, and S7). *tet*(L) appeared to have been introduced into the ancestral strain of the expanded ST2250 clone in Thailand and was present in all but five ST2250 isolates (Fig. 2A, S5, and S7). This gene has been frequently identified on plasmids derived from livestock-associated methicillin-resistant *S. aureus* (MRSA) belonging to ST398, as well as ST9 (22–24).

10.1128/mBio.00802-17.7 FIG S7  (A) Distribution of known antibiotic resistance genes across the phylogenetic tree. (B) Distribution of the variants of beta-lactamase Z genes in the ResFinder database across the phylogenetic tree. The red and light blue backgrounds of clades denote *S. aureus* and *S. argenteus*, respectively. Horizontal bars show gene presence (red) or absence (blue). Download FIG S7, PDF file, 0.2 MB. Copyright © 2017 Moradigaravand et al. 2017 Moradigaravand et al. This content is distributed under the terms of the Creative Commons Attribution 4.0 International license.

We explored the context of *tet*(L) and *bla*(Z) and found both genes located on a novel plasmid that did not show full sequence identity to any other previously reported elements and is named P1-tet(L)-cad-bla (Fig. 3D). This was 26.7 kbp in size and carried multiple antibiotic and heavy metal resistance-encoding genes, including *czcD*, which encodes an inducer of cobalt, copper, and cadmium resistance, and *acr*, which encodes arsenic resistance (Fig. 3D). Resistance to heavy metals in livestock-associated *S. aureus* has been frequently reported (25). Plasmid P1-tet(L)-cad-bla was most closely related to p18813-P04 (99% sequence identity across 88% of the sequence; GenBank accession number CP002146), which was derived from a human clinical isolate representing MRSA clone USA300 (26). Plasmids P1-tet(L)cad-bla and p18813-P04 varied on the basis of a region containing a cluster of antimicrobial resistance genes (Fig. 3D); i.e., plasmid p18813-P04 carries a *mer* operon that is absent from P1-tetL-cad-bla. Instead, P1-tet(L)-cad-bla contained a *tet*(L) resistance gene flanked by two copies of the IS*431* insertion sequence (Fig. 3D). Insertion elements such IS*431* have been previously implicated in the emergence of novel multiresistance plasmids by mediating plasmid cointegration (23). Several non-ST2250 isolates (ST1223) also carried an element that closely resembled the P1-tetL-cad-bla plasmid but lacked *tetL*, which we termed plasmid P2-cad-bla (Fig. 3D and S5). Plasmid P1-tet(L)-cad-bla shared 68% of its sequence with P2-cad-bla plasmid (99% identity), and was distinct from P2-cad-bla on the basis of the presence of *tet*(L), as well as the *czcD* and *acr3* heavy metal resistance genes (Fig. 3D). Similar to *tet*(L), the *aph*(H) gene appears to be more frequent in *S. argenteus* than in *S. aureus* (Fig. 3C) and is inserted in the chromosome (results not shown).

## DISCUSSION

In this study, we conducted an in-depth genomic comparison of community-acquired invasive *S. argenteus* (predominantly ST2250) and *S. aureus* isolates from people living in northeastern Thailand. The sampling framework, combined with the fine-scale resolution of whole-genome sequencing, allowed us to elucidate the differences and similarities between the genome contents of these closely related staphylococcal species. We found that ST2250 was the predominant ST of *S. argenteus* isolates and has become disseminated across northeastern Thailand. Furthermore, we found distinctive genomic features in ST2250 and several lines of evidence supporting gene flow or shared gene reservoirs between ST2250 and *S. aureus* plasmids and lineages.

Genes that were unique to ST2250 may indicate a biological basis for the success of this lineage based on the ability to be transmitted to, be carried by, or infect the human host. This contrasts with initial genomic and nongenomic findings on *S. argenteus*, which, on the basis of the lack of well-characterized *S. aureus* virulence factors such as staphyloxanthin, was considered to be a less virulent ancestor of *S. aureus* (9). Despite lacking several known *S. aureus* virulence factors, ST2250 has acquired putative virulence mechanisms that may have transformed ST2250 into an invasive human pathogen.

Our results also indicate that adaptation of ST2250 to ecological niches has occurred concurrently with the gain of genes that facilitate adaptation both within ST2250 [for example, the acquisition of *tet*(L)] and between ST2250 and other *S. argenteus* and *S. aureus* STs (for example, the acquisition of multiple enterotoxins in ST2250). In particular, *S. argenteus* has acquired genes previously observed only in livestock-associated *S. aureus* and plasmids. *S. argenteus* is frequently reported in remote populations (2, 5–7) where exposure to livestock is common, and our finding suggests that gene flow between livestock-associated *S. aureus* and *S. argenteus* has taken place. In the case of *tet*(L) and heavy metal resistance genes, the expansion of ST2250 appears to have occurred after the insertion of this gene into a plasmid in the ancestral strains, presumably in response to the use of this antibiotic in humans or animals. The impact of the gain of these genes in facilitating the adaptation and spread of ST2250 requires experimental verification. Sampling of animals in the region to isolate livestock-associated *S. aureus*, determine the presence of *S. argenteus*, and undertake a genomic comparison of isolates that cocolonize individual animals or farms would provide more direct evidence of genetic interaction between the bacterial species.

Our findings provide population genomic evidence that supports the genetic distinction between *S. argenteus* and *S. aureus*, with around half of the core genes being exclusive to each species. Despite this clear separation, convergent forces have led the two species to acquire similar virulence mechanisms and for *S. argenteus* to gain specific resistance and virulence genes from other *S. aureus* strains. Crucial to this are the mobile genetic elements and plasmids that have transferred important genetic elements between staphylococcal strains. Horizontal gene transfer has the potential to facilitate adaptation not only within one species (27) but also between two closely related species.

The deep-sampling method used here allowed us to obtain a high-resolution view of invasive *S. argenteus* ST2250 and to compare this with invasive *S. aureus* in the same population. Despite this, our study has several limitations that could be addressed in future studies to fully elucidate the global structure of the *S. argenteus* population and putative reservoirs. Most importantly, our study was focused on one region that was dominated by a single *S. argenteus* ST. Furthermore, our collection was restricted to invasive isolates associated with community onset infection and therefore does not fully represent the whole population associated with carriage or with hospital-associated infection, including outbreaks, for which denser sampling and comprehensive epidemiological data would be required. A study of global *S. argenteus* and assessment of its presence in livestock are now warranted.

## MATERIALS AND METHODS

### Isolate collection.

The isolate collection from northeastern Thailand consisted of 251 *S. aureus* and 68 *S. argenteus* isolates drawn from two prior studies (8). The first of these contributed 10 *S. argenteus* isolates from patients with community onset invasive staphylococcal diseases at Sunpasitthiprasong Hospital, Ubon Ratchathani, between 2006 and 2007 (8). The second study contributed 251 *S. aureus* and 58 *S. argenteus* isolates from patients recruited into an observational study of community onset invasive staphylococcal infections conducted at four hospitals across northeastern Thailand between 2010 and 2013 (8). In brief, patients were identified in both studies by daily screening at each hospital diagnostic microbiology laboratory. An invasive infection was defined as culture of the isolate from a sterile-site sample. A community onset infection was defined as a positive culture taken within 2 days of hospital admission. Ethical approval for the 2006-2007 study was obtained from the Ethical and Scientific Review subcommittee of the Royal Thai Government Ministry of Public Health and the Oxford Tropical Research Ethics Committee. Ethical approval for the 2010 to 2013 study was obtained from the Faculty of Tropical Medicine, Mahidol University (approval no. MUTM 2011-007-01); Sunpasitthiprasong Hospital, Ubon Ratchathani (approval no. 004/2553); Udon Thani Hospital, Udon Thani (approval no. 0027.102/2349); Khon Kaen Hospital, Khon Kaen; and Faculty of Medicine (Srinagarind Hospital), Khon Kaen University, Khon Kaen, Thailand (approval no. HE541113).

Antibiotic susceptibility data for the collection had been established previously for cefoxitin, clindamycin, trimethoprim-sulfamethoxazole, erythromycin, fosfomycin, fusidic acid, gentamicin, levofloxacin, oxacillin, penicillin, tigecycline, and vancomycin (for 10 isolates, fosfomycin, levofloxacin, and tigecycline susceptibility data were not available). All *S. argenteus* isolates were methicillin susceptible and *mecA* negative, while seven *S. aureus* isolates (six ST2399 isolates and one ST241 isolate) were MRSA and *mecA* positive. *pvl* was detected in 13% (9/68) of the *S. argenteus* isolates. MLST performed previously revealed 38 STs in total. The most common STs of *S. aureus* and *S. argenteus* were ST121 (*n* = 108, 42%) and ST2250 (*n* = 57, 83%), respectively.

To evaluate the Thai *S. argenteus* ST2250 isolates in relation to *S. argenteus* isolates obtained from other geographic regions, additional genomes were obtained for a further 10 clinical isolates (6 from Malaysia, 2 from Singapore, and 1 each from France and Israel), obtained from the Tigecycline Evaluation and Surveillance Trial between 2009 and 2011 (28). The accession numbers and the attributes of the isolates studied are provided in Table S1.

10.1128/mBio.00802-17.8 TABLE S1  Accession numbers and metadata of the isolates studied. Download TABLE S1, CSV file, 0.04 MB. Copyright © 2017 Moradigaravand et al. 2017 Moradigaravand et al. This content is distributed under the terms of the Creative Commons Attribution 4.0 International license.

### Sequencing and pangenome analysis.

Genomic DNA was extracted with the QIAxtractor (Qiagen) as detailed in the manufacturer’s instructions. Illumina sequencing libraries with a 450-bp insert size were prepared in accordance with the manufacturer’s protocol and sequenced on an Illumina HiSeq2000 with 100-bp paired-end reads. Reads were mapped to the reference genome of *S. aureus* (strain NCTC 8325) for *S. aureus* isolates and tht of *S. argenteus* (strain MSHR1132) for *S. argenteus* isolates with SMALT v0.7.4 (www.sanger.ac.uk/science/tools/smalt-0) by using maximum and minimum inserts sizes of 1,000 and 50, respectively. SNPs were then called and annotated with SAMtools mpileup (29) and BCFtools as detailed in reference 30. The parameter values included a minimum base call quality of 50 and a minimum root mean squared mapping quality of 30 to call an SNP. SNPs at sites with heterogeneous mapping, where the SNP was present in <75% of the reads, were removed. The average mapping quality was 84% (minimum, 77%; maximum, 93%) for *S. aureus* isolates and 93% (minimum, 92%; maximum, 94%) for *S. argenteus* isolates.

We then used an assembly improvement pipeline (31) based on Velvet (32) to create *de novo* assemblies from short reads. Assemblies were annotated with Prokka (33), the output of which was used as input for the pangenome pipeline Roary (34) by using the default parameter values, including a minimum percent identity of 95%. Roary produced an alignment of core genes. The output of Roary and sequences in the pangenome of the entire collection have been deposited in a public repository (https://data.mendeley.com/datasets/phphbtzjxh/2). SNPs in the core genome alignment were identified with an in-house tool that is publicly available at http://www.github.com/sanger-pathogens/snp-sites. We identified 123,850 SNP sites in the core genome alignment of the *S. aureus* and *S. argenteus* isolates. Neighbor-joining phylogenetic trees were constructed with the ape package in R. Microreact (http://www.microreact.org), iTOL (35), FigTree (http://tree.bio.ed.ac.uk/software/figtree/), and Easyfig (36) were used to visualize the tree and the associated metadata.

HierBAPS (37) was used to conduct BAPS to cluster similar genomic sequences, for which SNPs were first extracted from the multiple alignment of the *S. aureus* and *S. argenteus* core genomes with an in-house tool (available at http://www.github.com/sanger-pathogens/snp-sites) and the parameters of two clustering iterations and expected numbers of clusters of 20, 40, and 60. We considered BAPS clusters to represent recent clades, and these corresponded to clades comprising individual STs in the *S. aureus* collection. The *S. argenteus* ST2250 clade was created by merging two BAPS clusters. These recent clades were then used in downstream Bayesian analysis.

### Identification of antimicrobial resistance genes.

Known antibiotic resistance genes were identified with the SRST2 package by using a 90% coverage cutoff (38). For specific accessory genes of interest, we determined whether they were associated with a mobile genetic element. To do this, we identified all accessory elements, which were defined as genomic fragments not uniformly distributed across all of the isolates analyzed, as described previously (30). We performed a BLAST analysis of genes of interest against the identified accessory elements to determine their association. We then ran BLAST on the whole identified mobile genetic element against the NCBI nonredundant nucleotide database to determine whether the mobile genetic element exhibited similarity to previously sequenced genomes. To find the distribution of the mobile genetic element across the other isolates analyzed, we used MUMmer (39) to map the whole-genome assemblies against the mobile genetic elements identified. Reads were then mapped to the reference genomes of plasmids as detailed above.

### Recombination analysis and Bayesian analysis.

After identifying recent clades on the phylogenetic tree, we extracted isolates within each clade and mapped the reads to the local reference pseudogenome constructed by concatenating contigs of the isolate with the highest N50 value (the best assembly statistics). The mapping and SNP calling and annotation were done as described above.

After constructing the multiple alignment for each recent clade, we identified potential recombination as high SNP density blocks with Gubbins (11) by using five iterations. In total, we found 117 recombinant blocks (average size, 12,551 bp; minimum, 22 bp; maximum, 46,558 bp) and extracted the genomic regions longer than 100 bp to identify potential donors of the recombined regions by searching the NCBI nonredundant nucleotide data set with BLAST. To obtain the recent substitution rate and divergence times of the clusters identified in the population, recombined regions were removed and the alignment was used as the input for the downstream Bayesian analysis.

To assess the temporal signal of the major clades, ST88, ST6, ST97, ST1, ST121, and ST2250, we assessed the significance of the R-squared value from the plot of root-to-tip distance versus time of isolation. To this end, we first constructed a neighbor-joining tree from the alignment for each clade and plotted the root-to-tip distance values against the years of isolation. After extracting the R-squared value, we generated 10,000 samples by randomizing the years of isolation (i.e., resampling with replacement) and then assessed the value of the real R-squared value against the distribution of R-squared values. We found a strong signal for the ST2250 clade (the R-squared value of the data set was >99% of the calculated R-squared values for randomized samples) and weaker signals (the R-squared value of the data set was >60% of the calculated R-squared values for randomized samples) for the ST121 and ST6 clades (Fig. S2A to C). ST88, ST1, and ST97 lacked any temporal signal and therefore were excluded from the Bayesian analysis (Fig. S2C).

We then performed a Bayesian analysis within BEAST v1.7 (12) on clades with a temporal signal, i.e., clades ST2250, ST121, and ST6, testing various models that included a constant population size with a strict molecular clock (with uniform, normal, and log-normal distributions). Furthermore, continuous phylogeography analysis of the Thai isolates within the ST121, ST6, and ST2250 clades was performed with BEAST (by using the same models as above) and by incorporating the longitude and latitude information for patient addresses in the model. In each run, we ran three independent chains for 50 million generations, sampling every 10 generations. We then excluded 10 million initial states as burn-in and used an effective sample size cutoff value of >200. The TreeAnnotator software, which is part of the BEAST package, was used to summarize the trees and obtain CIs for divergence times, node ages, and node estimated locations.

To assess whether improving the temporal signal influences the results for the ST121 clade, which is the most common *S. aureus* ST in our collection, we included a further six global ST121 isolates from reference 13 that were mapped >90% to the reference genome. The inclusion of these isolates resulted in a strong temporal signal (the R-squared value of the data set was >99% of the calculated R-squared values for randomized samples) (Fig. S2B). Although this resulted in smaller 95% CIs for the key parameters, such as the age of the MRCA and substitution rates, the results remained similar, indicating that the temporal signal within the Thai isolates appears to be robust.

To compute the age of the MRCA of the *S. aureus* clades without a temporal signal, i.e., the ST88, ST97, and ST1 clades, we divided the root distance of each tree by the mean substitution rates of the ST121 and ST6 clades, which had temporal signals. In addition, we used the means of the maximum and minimum values of the upper and lower 95% error bars of the substitution rates of the ST121, ST6, and ST2250 clades to compute the error bars for the ST97, ST88, and ST1 clades.

We used the maximum-likelihood tool in the phytools package (40) to obtain the probabilities of the ancestral states of the country of origin. Since sampling bias (i.e., different sampling counts from different countries) can potentially affect the findings on the ancestral status of the root, we conducted a sensitivity analysis to determine the impact of the size of the Thai collection on the inferred status of the root. To this end, we constructed collections consisting of non-Thai isolates and added a random subsample of Thai isolates. We then calculated the marginal likelihood of the root of the constructed trees. We repeated this step 50 times for six sample sizes and report the mean and standard deviation of each sample size (Fig. S3).

### Data availability.

The sequence data obtained in this study have been submitted to the European Nucleotide Archive (http://www.ebi.ac.uk/ena) under the accession numbers listed in Table S1. The study numbers are PRJEB9575 (http://www.ebi.ac.uk/ena/data/view/PRJEB9575) and PRJEB1915 (http://www.ebi.ac.uk/ena/data/view/PRJEB1915) for the Thai and non-Thai collections, respectively.
