# Supplementary figures and images for: Evolution of the Staphylococcus argenteus ST2250 Clone in Northeastern Thailand Is Linked with the Acquisition of Livestock-Associated Staphylococcal Genes
Source: mBio. 2017 Jul 5;8(4):e00802-17. doi: 10.1128/mBio.00802-17 (PMC5573676; doi:10.1128/mBio.00802-17)

**A**

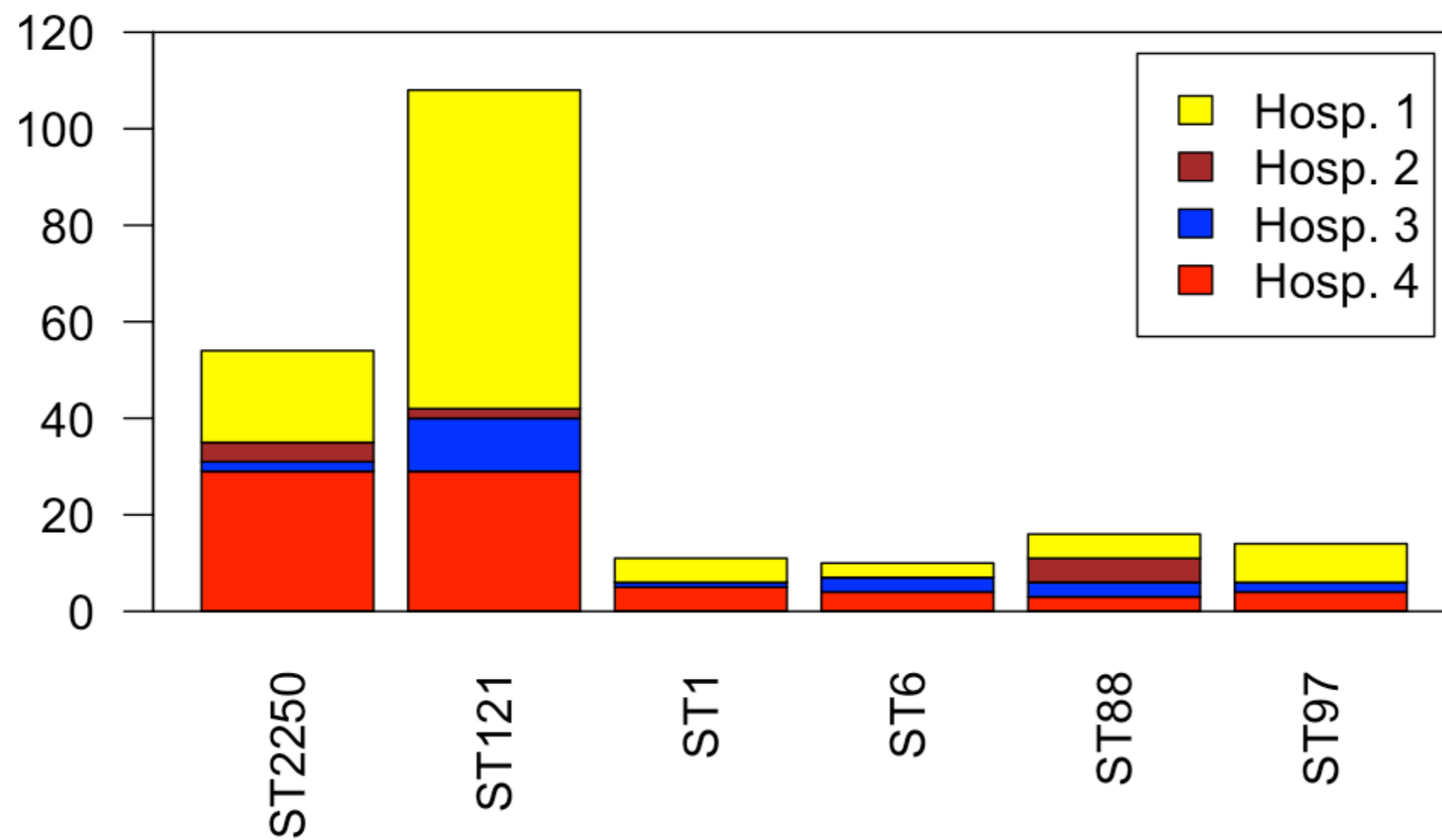

**B**

**ST2250**

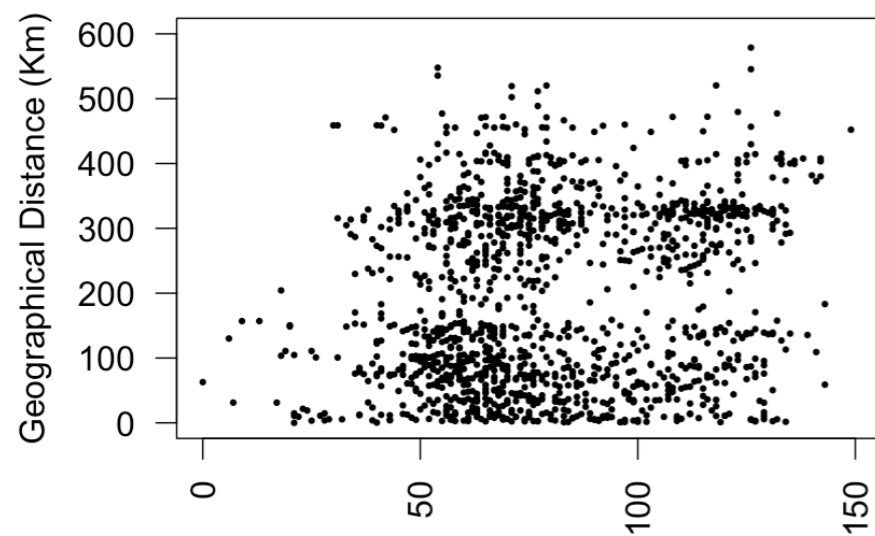

**ST88**

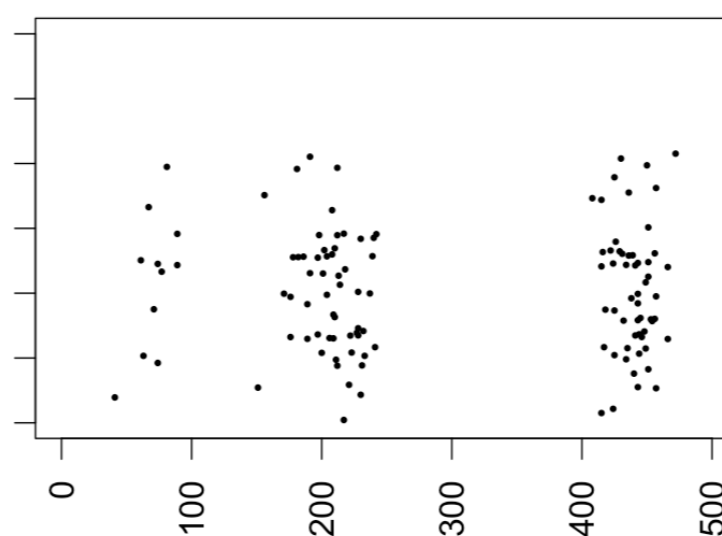

**ST97**

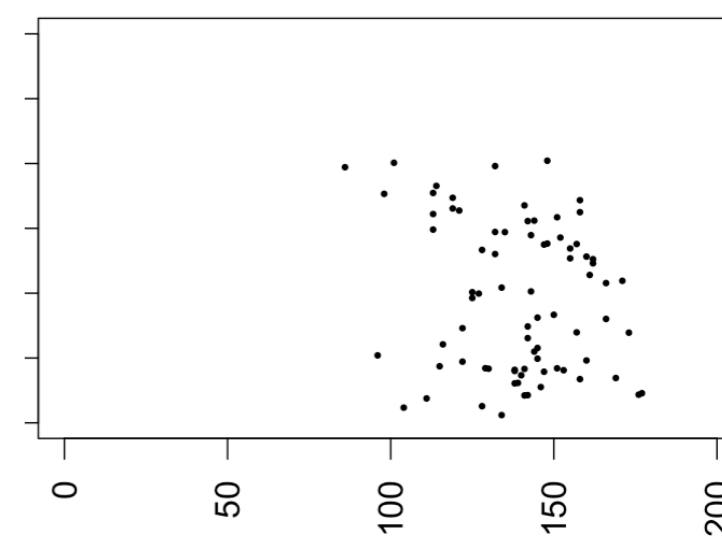

**ST121**

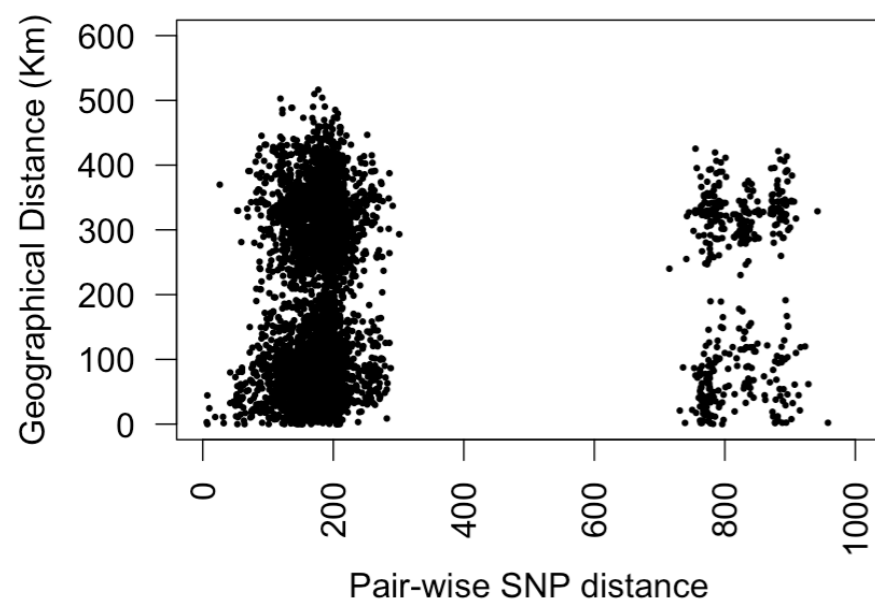

**ST6**

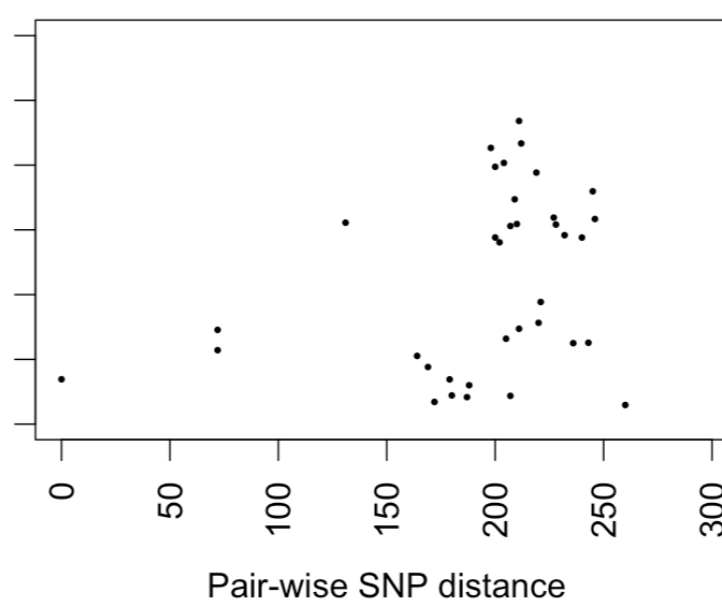

**ST1**

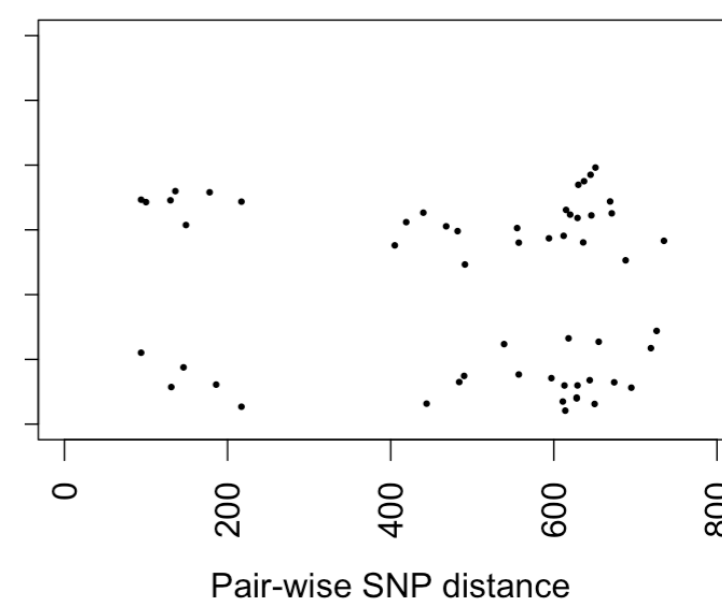

Supplement: FIG S1 [file mbo003173374sf1.pdf]

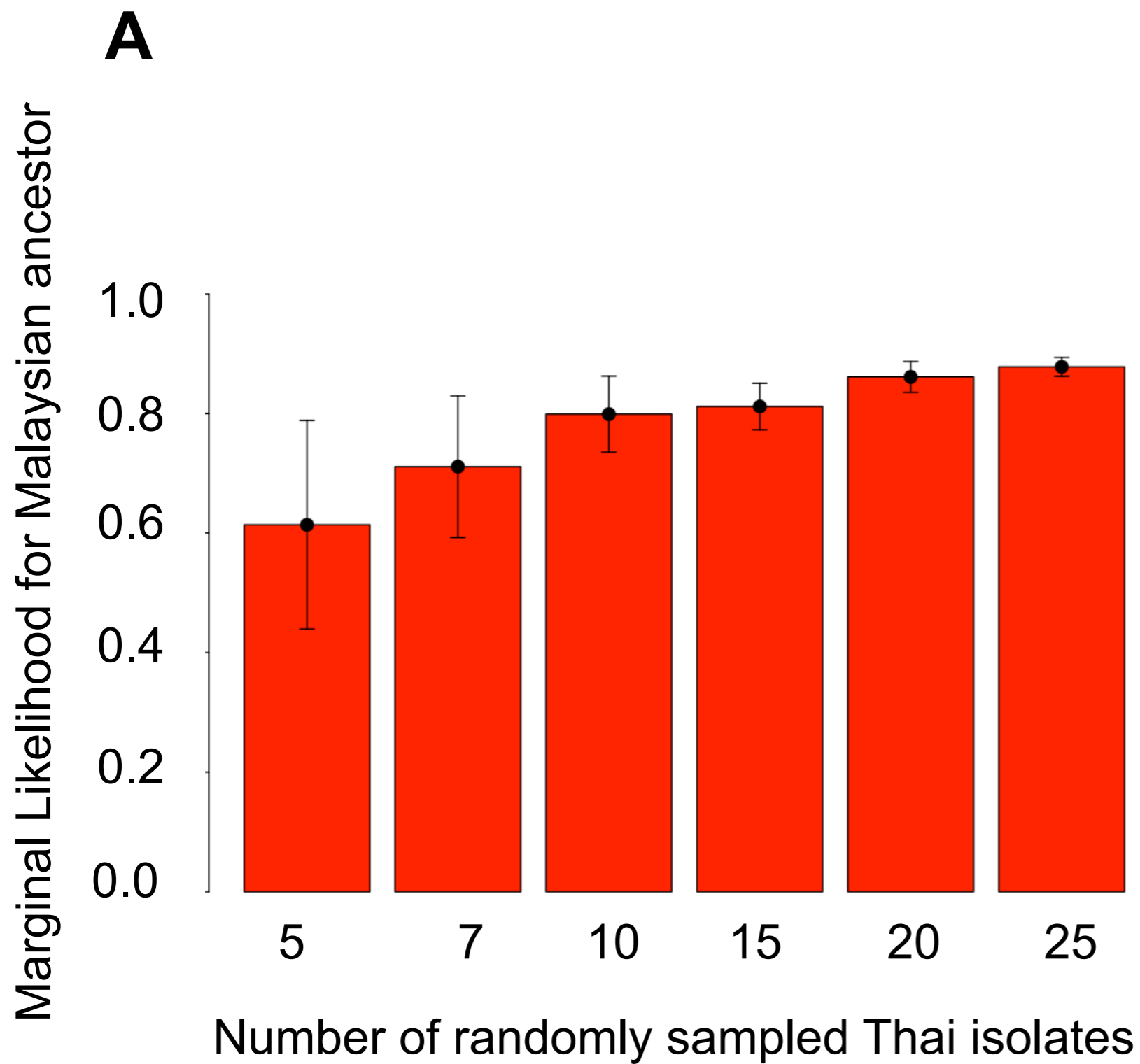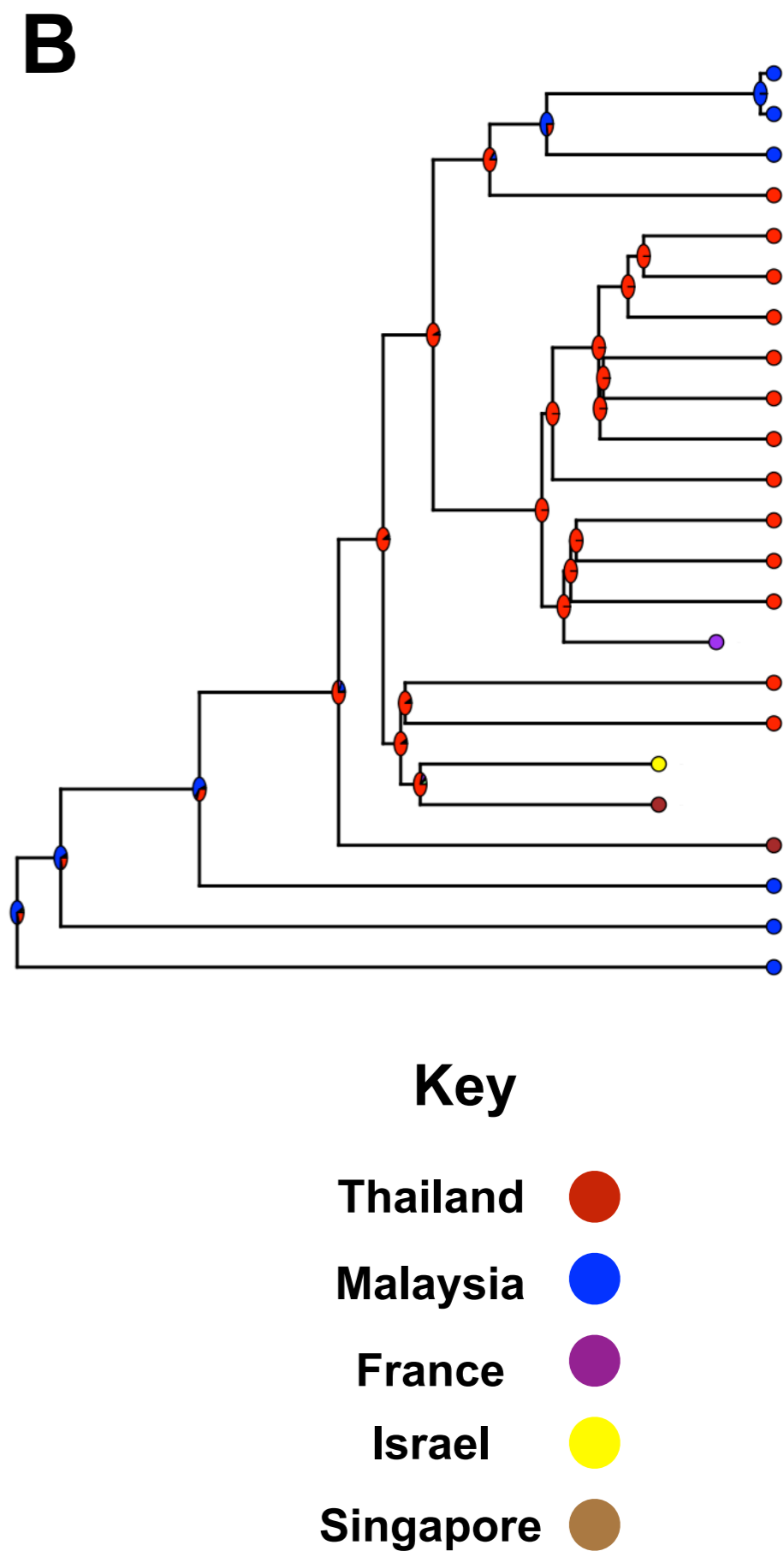

Supplement: FIG S3 [file mbo003173374sf3.pdf]
