# Supplementary material for: Evolution of the Staphylococcus argenteus ST2250 Clone in Northeastern Thailand Is Linked with the Acquisition of Livestock-Associated Staphylococcal Genes
Source: mBio. 2017 Jul 5;8(4):e00802-17. doi: 10.1128/mBio.00802-17 (PMC5573676; doi:10.1128/mBio.00802-17)
Supplement: FIG S2 [file mbo003173374sf2.pdf]

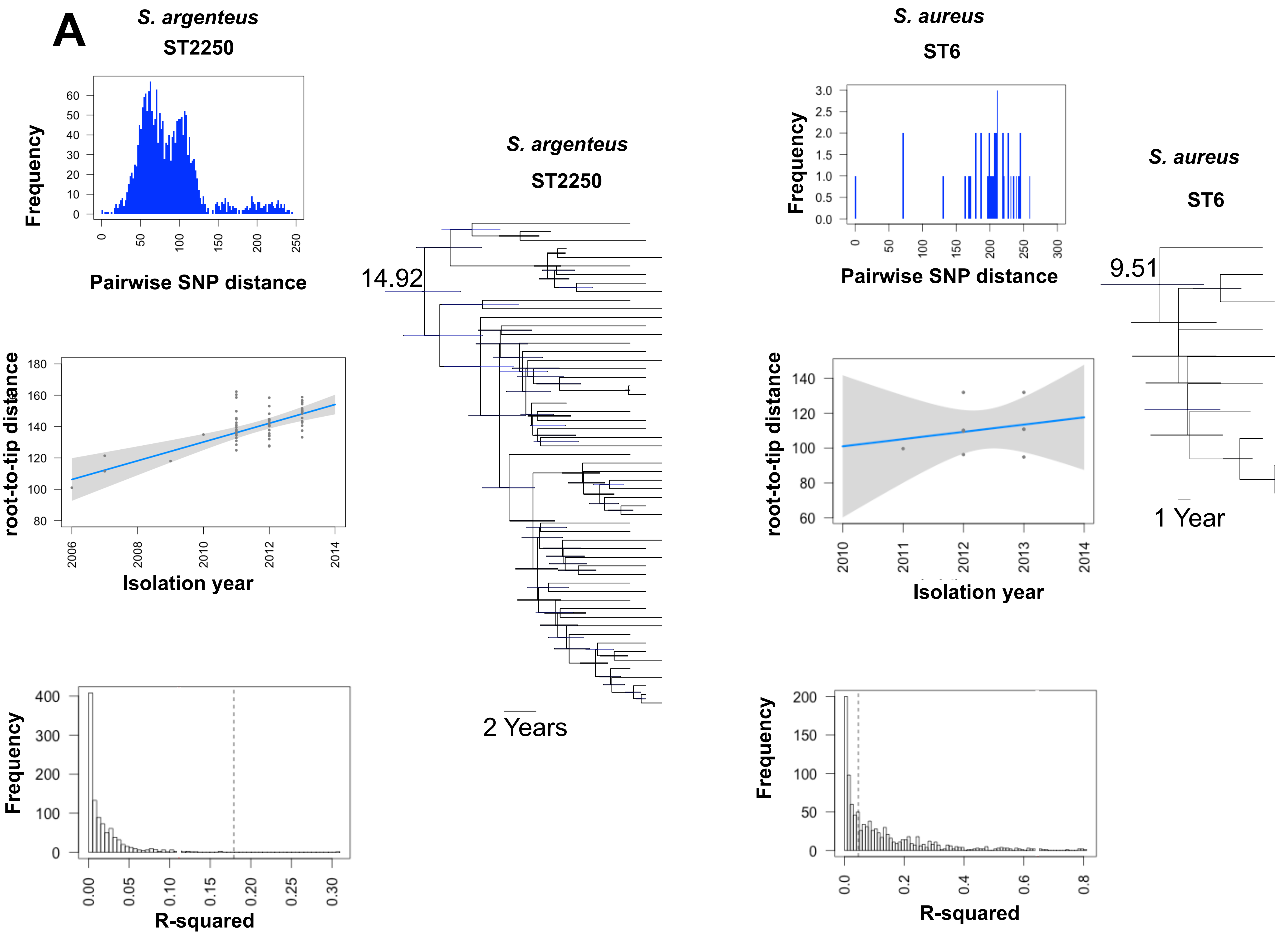
A
S. argenteus
ST2250
Frequency
Pairwise SNP distance
root-to-tip distance
Isolation year
R-squared
S. argenteus
ST2250
14.92
2 Years
S. aureus
ST6
Frequency
Pairwise SNP distance
S. aureus
ST6
9.51
1 Year
root-to-tip distance
Isolation year
Frequency
R-squared

B

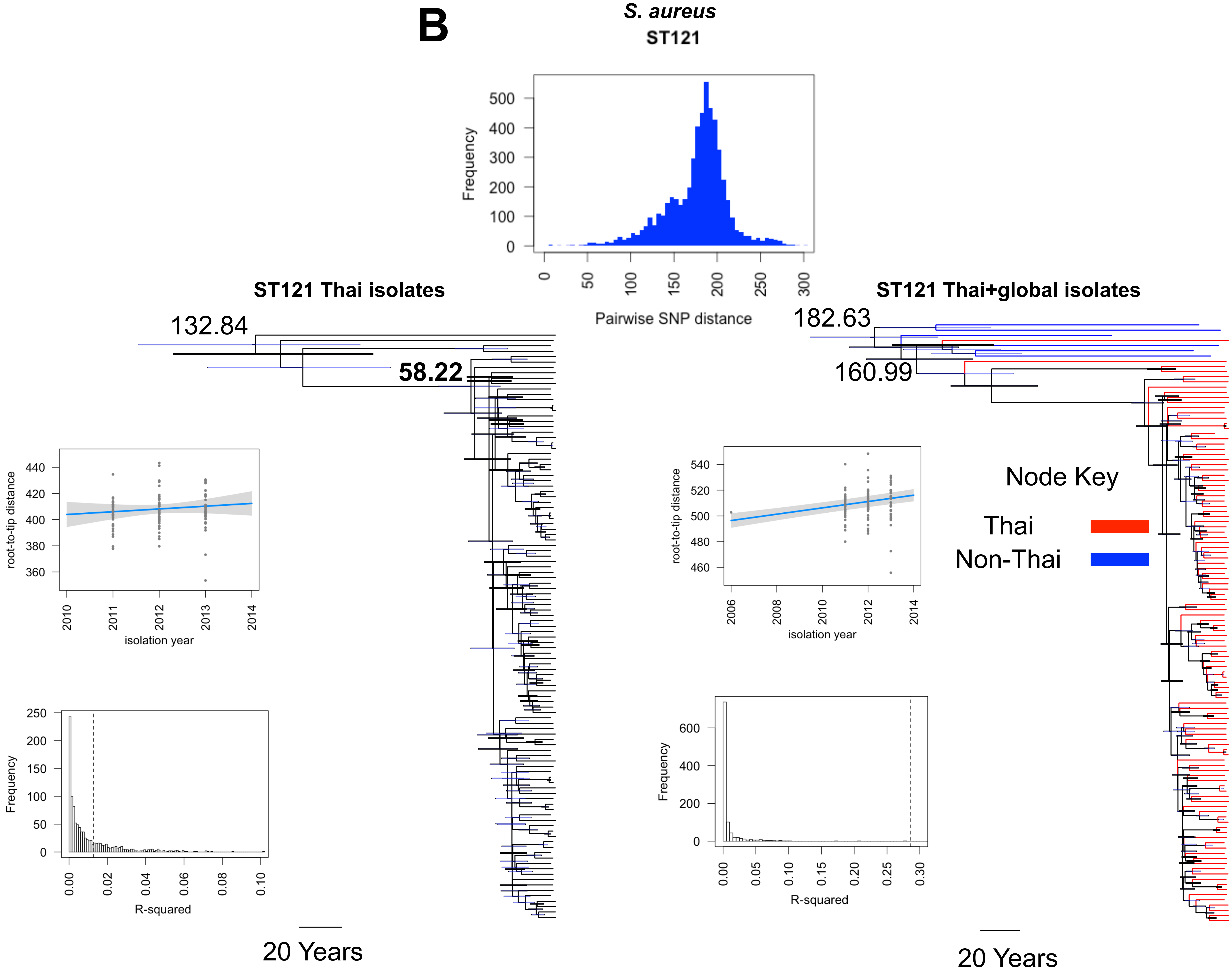

S. aureus
ST121
Frequency
500
400
300
200
100
0
0
50
100
150
200
250
300
Pairwise SNP distance
ST121 Thai isolates
132.84
58.22
root-to-tip distance
440
420
400
380
360
2010
2011
2012
2013
2014
isolation year
Frequency
250
200
150
100
50
0
0.00
0.02
0.04
0.06
0.08
0.10
R-squared
20 Years
ST121 Thai+global isolates
182.63
160.99
root-to-tip distance
540
520
500
480
460
2006
2008
2010
2012
2014
isolation year
Node Key
Thai
Non-Thai
Frequency
600
400
200
0
0.00
0.05
0.10
0.15
0.20
0.25
0.30
R-squared
20 Years

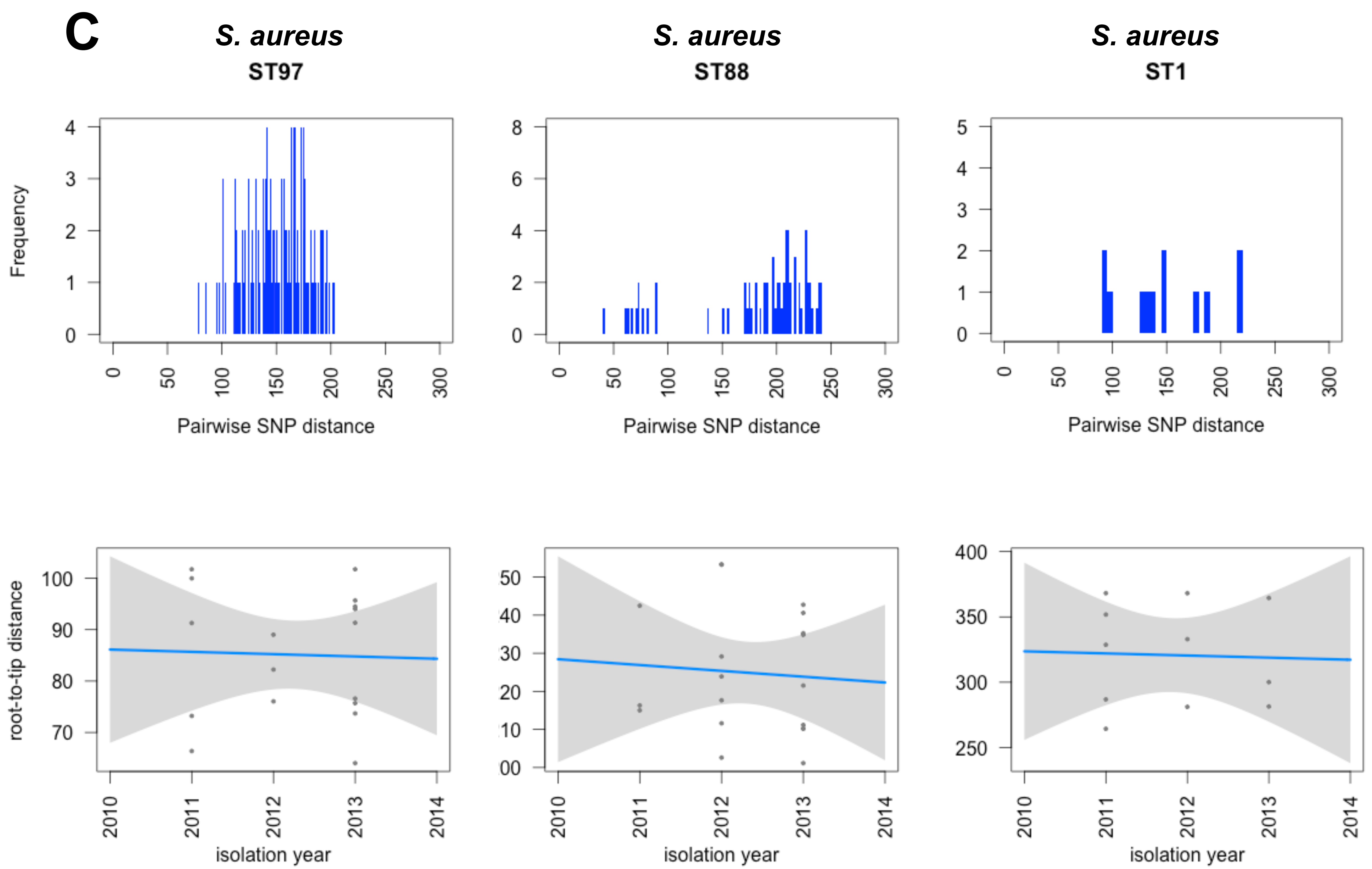
C
S. aureus
ST97
S. aureus
ST88
S. aureus
ST1
Frequency
Pairwise SNP distance
Pairwise SNP distance
Pairwise SNP distance
root-to-tip distance
isolation year
isolation year
isolation year
