# Supplementary material for: Evolution of the Staphylococcus argenteus ST2250 Clone in Northeastern Thailand Is Linked with the Acquisition of Livestock-Associated Staphylococcal Genes
Source: mBio. 2017 Jul 5;8(4):e00802-17. doi: 10.1128/mBio.00802-17 (PMC5573676; doi:10.1128/mBio.00802-17)
Supplement: FIG S4 [file mbo003173374sf4.pdf]

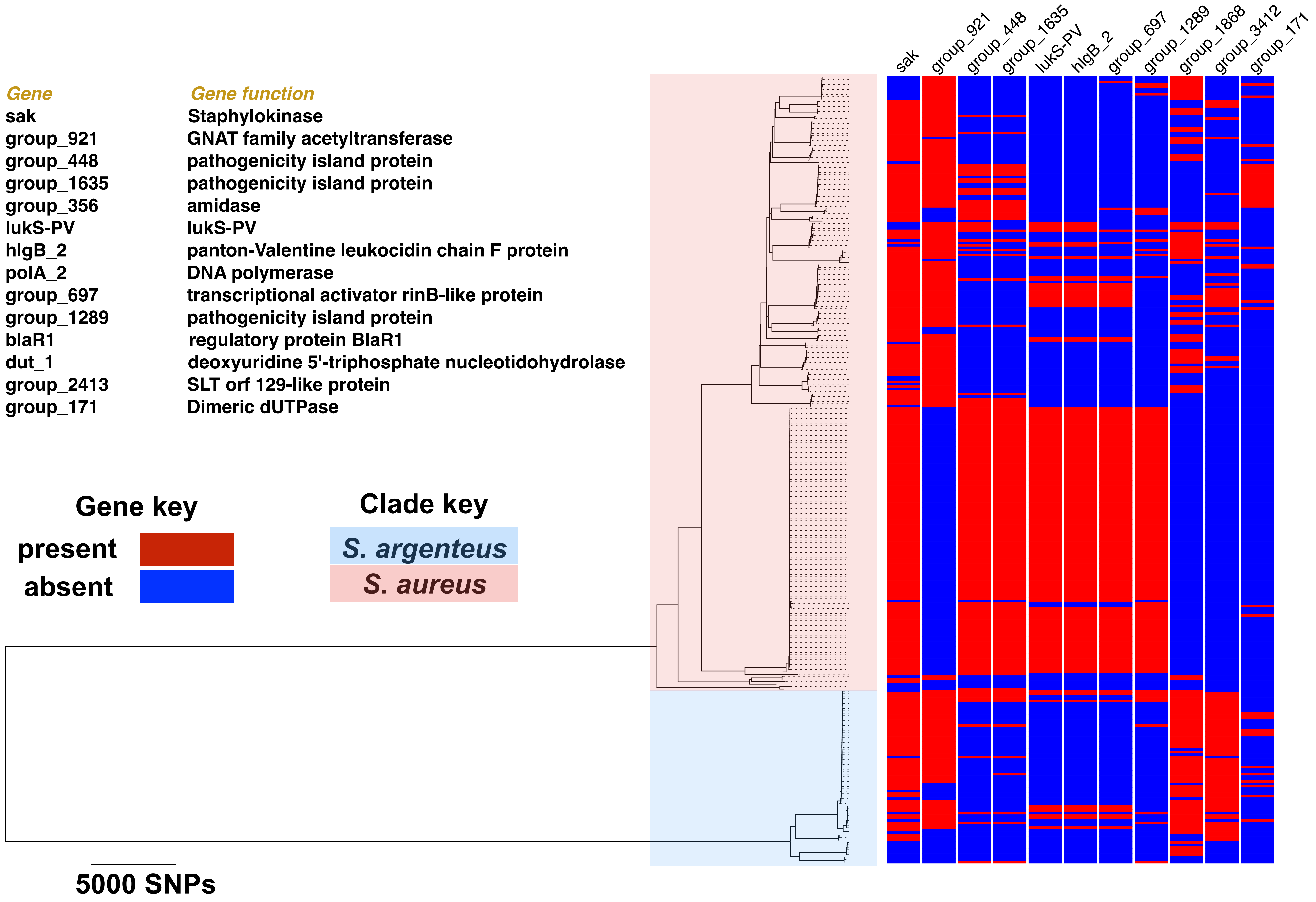
sak
group_921
group_448
group_1635
lukS-PV
hlgB_2
group_697
group_1289
group_1868
group_3412
group_171
Gene
Gene function
sak
Staphylokinase
group_921
GNAT family acetyltransferase
group_448
pathogenicity island protein
group_1635
pathogenicity island protein
group_356
amidase
lukS-PV
lukS-PV
hlgB_2
panton-Valentine leukocidin chain F protein
polA_2
DNA polymerase
group_697
transcriptional activator rinB-like protein
group_1289
pathogenicity island protein
blaR1
regulatory protein BlaR1
dut_1
deoxyuridine 5'-triphosphate nucleotidohydrolase
group_2413
SLT orf 129-like protein
group_171
Dimeric dUTPase
Gene key
present
absent
Clade key
S. argenteus
S. aureus
5000 SNPs
