# Supplementary material for: Evolution of the Staphylococcus argenteus ST2250 Clone in Northeastern Thailand Is Linked with the Acquisition of Livestock-Associated Staphylococcal Genes
Source: mBio. 2017 Jul 5;8(4):e00802-17. doi: 10.1128/mBio.00802-17 (PMC5573676; doi:10.1128/mBio.00802-17)
Supplement: FIG S5 [file mbo003173374sf5.pdf]

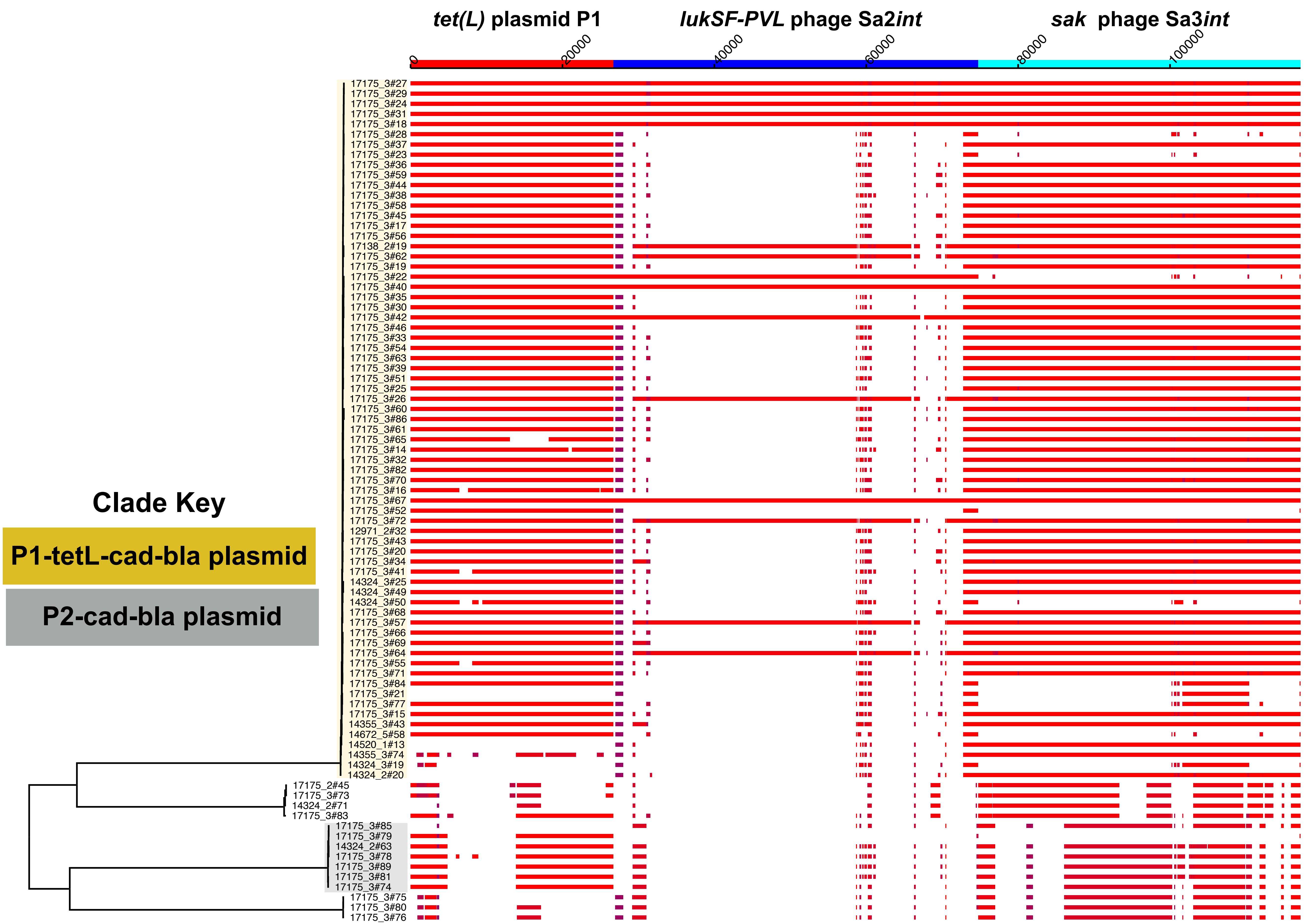

tet(L) plasmid P1
lukSF-PVL phage Sa2int
sak phage Sa3int
0
20000
40000
60000
80000
100000
Clade Key
P1-tetL-cad-bla plasmid
P2-cad-bla plasmid
17175_3#27
17175_3#29
17175_3#24
17175_3#31
17175_3#18
17175_3#28
17175_3#37
17175_3#23
17175_3#36
17175_3#59
17175_3#44
17175_3#38
17175_3#58
17175_3#45
17175_3#17
17175_3#56
17138_2#19
17175_3#62
17175_3#19
17175_3#22
17175_3#40
17175_3#35
17175_3#30
17175_3#42
17175_3#46
17175_3#33
17175_3#54
17175_3#63
17175_3#39
17175_3#51
17175_3#25
17175_3#26
17175_3#60
17175_3#86
17175_3#61
17175_3#65
17175_3#14
17175_3#32
17175_3#82
17175_3#70
17175_3#16
17175_3#67
17175_3#52
17175_3#72
12971_2#32
17175_3#43
17175_3#20
17175_3#34
17175_3#41
14324_3#25
14324_3#49
14324_3#50
17175_3#68
17175_3#57
17175_3#66
17175_3#69
17175_3#64
17175_3#55
17175_3#71
17175_3#84
17175_3#21
17175_3#77
17175_3#15
14355_3#43
14672_5#58
14520_1#13
14355_3#74
14324_3#19
14324_2#20
17175_2#45
17175_3#73
14324_2#71
17175_3#83
17175_3#85
17175_3#79
14324_2#63
17175_3#78
17175_3#89
17175_3#81
17175_3#74
17175_3#75
17175_3#80
17175_3#76
