# Supplementary material for: Evolution of the Staphylococcus argenteus ST2250 Clone in Northeastern Thailand Is Linked with the Acquisition of Livestock-Associated Staphylococcal Genes
Source: mBio. 2017 Jul 5;8(4):e00802-17. doi: 10.1128/mBio.00802-17 (PMC5573676; doi:10.1128/mBio.00802-17)

A

Gene	Gene Function
isdH_1	haptoglobin-binding surface protein
maeA_1	putative malolactic enzyme
group_2498	AEC family malonate efflux carrier
group_2507	Insertion sequenceATP-binding protein, putative
cas1	Cas1 family protein
csm5	Csm5 family CRISPR-associated RAMP protein
sec-bov	enterotoxin C-bovine
entQ	staphylococcal enterotoxin Q
group_5336	hypothetical protein with DUF1433
group_3632	CRISPR-associated protein
group_4883	coagulase family protein
group_4884	extracellular matrix and plasma binding protein
group_4957	putative lipoprotein
group_5213	FIG01108398: hypothetical protein
gdmA_1	lantibiotic protein
apr	subtilase family protease
group_5241	Doubtful CDS. No database matches
group_5339	putative lipoprotein
group_5350	virus attachment p12 family protein
group_5366	membrane protein
hsdR_3	type I restriction-modification system endonuclease (fragment)
hsdR_2	type I restriction-modification system endonuclease (fragment)
group_5423	type I restriction-modification system specificity protein (fragment)
cas2	CRISPR-associated protein Cas2
csm1	CRISPR-associated Csm1 family protein
csm2	CRISPR-associated Csm2 protein
csm3	Csm3 family CRISPR-associated RAMP protein
group_5428	CRISPR-associated Csm4 family protein
cas6	CRISPR-associated protein C
group_5430	putative reverse transcriptase
group_5431	hypothetical protein
group_5432	hypothetical protein
group_5433	hypothetical protein
group_5438	Membrane lipoprotein
group_5506	Repetitive hypothetical protein
group_5509	Putative cytosolic protein
group_5536	exotoxin
group_5537	exotoxin set-1..2…3.. on the same operon
group_5538	putative restriction and modification system specificity protein
group_5539	exotoxin
group_5540	putative surface protein
lpl2_2	Membrane lipoprotein
group_5545	putative lipoprotein
group_5546	lipase family protein

B

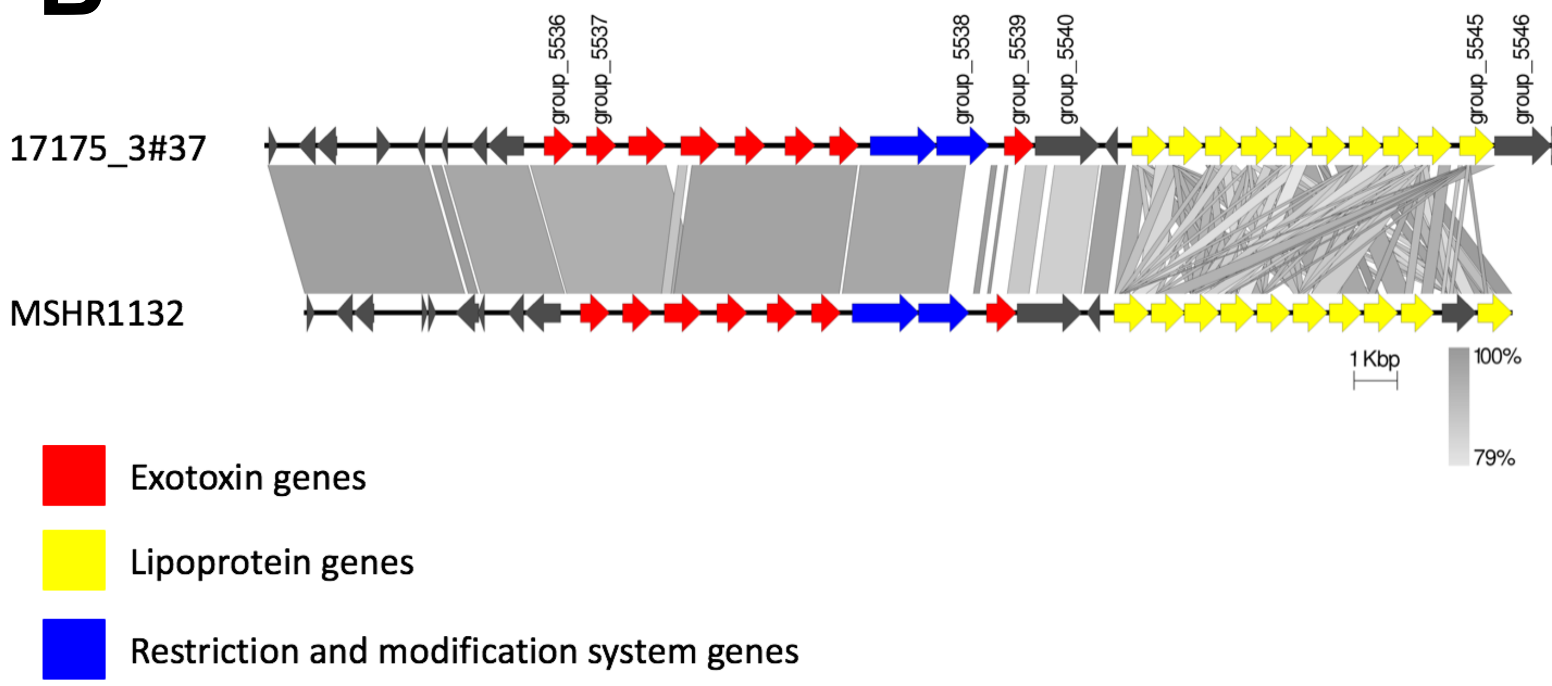

Supplement: FIG S6 [file mbo003173374sf6.pdf]
