# Supplementary material for: Evolution of the Staphylococcus argenteus ST2250 Clone in Northeastern Thailand Is Linked with the Acquisition of Livestock-Associated Staphylococcal Genes
Source: mBio. 2017 Jul 5;8(4):e00802-17. doi: 10.1128/mBio.00802-17 (PMC5573676; doi:10.1128/mBio.00802-17)
Supplement: FIG S7 [file mbo003173374sf7.pdf]

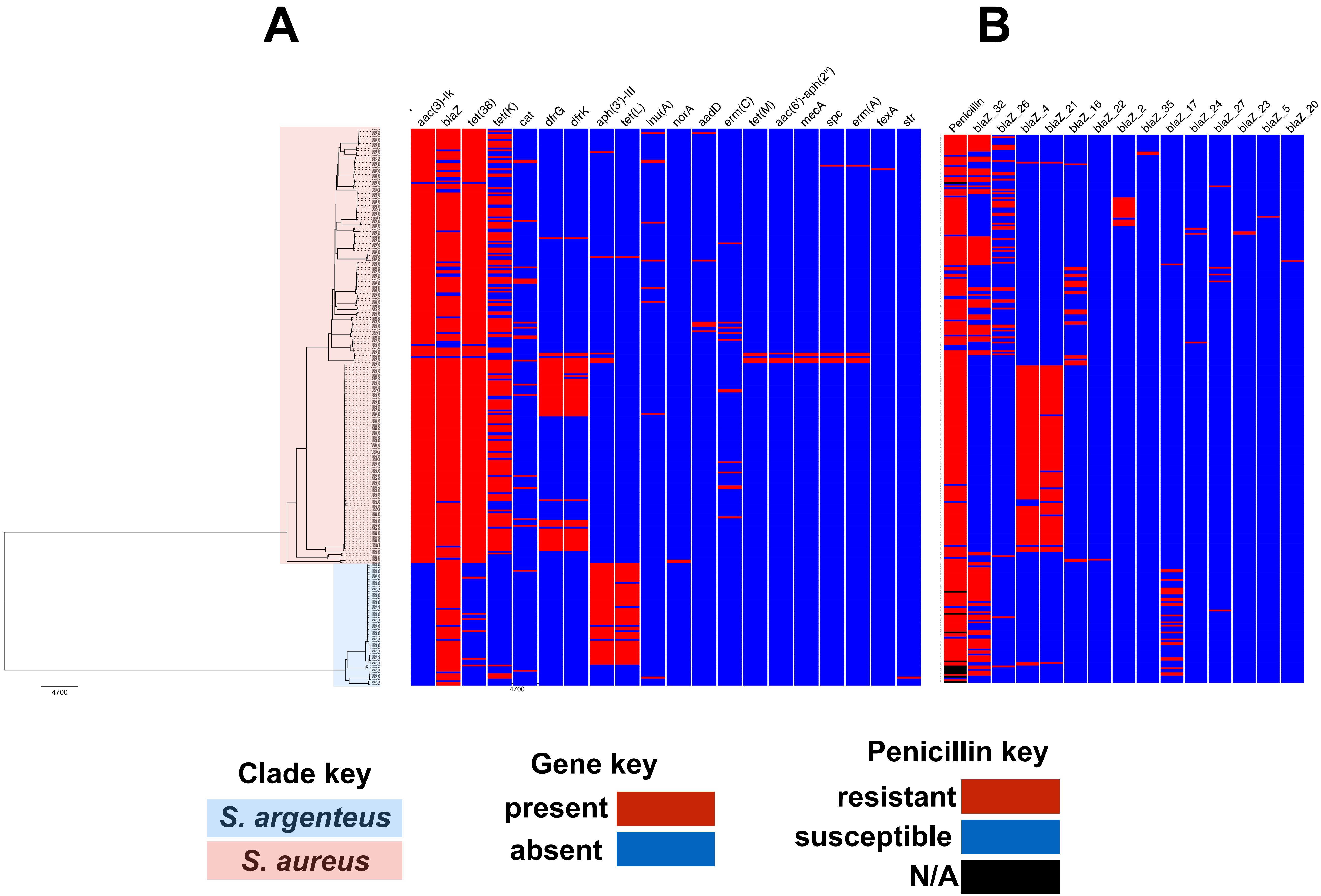

A
B
aac(3)-Ik
blaZ
tet(38)
tet(K)
cat
dfrG
dfrK
aph(3')-III
tet(L)
lnu(A)
norA
aadD
erm(C)
tet(M)
aac(6')-aph(2'')
mecA
spc
erm(A)
fexA
str
Penicillin
blaZ_32
blaZ_26
blaZ_4
blaZ_21
blaZ_16
blaZ_22
blaZ_2
blaZ_35
blaZ_17
blaZ_24
blaZ_27
blaZ_23
blaZ_5
blaZ_20
4700
4700
Clade key
S. argenteus
S. aureus
Gene key
present
absent
Penicillin key
resistant
susceptible
N/A
